# The relationship between smartphone usage duration (using smartphone’s ability to monitor screen time) with hand-grip and pinch-grip strength among young people: an observational study

**DOI:** 10.1186/s12891-021-04054-6

**Published:** 2021-02-15

**Authors:** Ahmad Osailan

**Affiliations:** Physical Therapy and Health Rehabilitation, College of Applied Medical Sciences, Prince Sattam bin Abdulaziz University, Riyadh Region 16237 Alkharj, Saudi Arabia

**Keywords:** Smartphones overuse, Hand-grip strength, pinch-grip strength, Hand functions

## Abstract

**Background:**

The use of smartphones has become widely popular, especially among young people, for multiple purposes other than communication, including gaming and internet browsing. The hand and wrist weakness is one of the main complications associated with the increased use of smartphones. This weakness occurs due to the repetitive flexion and extension of the wrist, thumb, and fingers, leading to a significant musculoskeletal pathology. Little is known about the relationship between smartphone usage duration (using the phones ability to monitor screen time) and hand-grip, pinch-grip strength. Therefore, the study was aimed to investigate the association between smartphone usage duration and hand-grip, pinch-grip strength among young people.

**Methods:**

One hundred young males volunteered to participate in the study. Participants were briefly examined for height and weight using a portable stadiometer and a digital scale. Hand-grip, pinch-grip strength measurement was performed using a hand-held dynamometer. Smartphones usage duration was obtained from the daily average screen time reported in the last seven days.

**Results:**

Mean daily usage of smartphone among the participants was 7.8 ± 2.2. There was a weak significant inverse relationship between smartphone usage duration and hand-grip strength (*r=*-.22, *p* = .03) and pinch-grip strength (*r*=-.28, *p* = .004). Linear regression revealed that 18.8 % of the variance in hand-grip strength and 20.4 % of the variance in pinch-grip strength was explained by age, and smartphone usage duration, with the addition of BMI only to hand-grip strength (*p’s* < 0.00).

**Conclusions:**

Prolonged use of smartphones was related to weaker hand-grip and pinch-grip. Despite the weak relationship, the study showed that smartphone usage duration might contribute as a factor along with age to hand muscles’ strength.

## Background

The use of smartphones has become a necessity for everyone in their daily life. Smartphones have been recently used for communication, gaming, socializing, and internet browsing, especially by the younger population. In the last few years, there has been a constant increase in the number of people using smartphones. In 2020, the number of smartphone users globally had projected to 3.5 billion, which is more by 9.3 % than in 2019 [1]. Also, it has been suggested by many surveys that younger adults are the ones who represent the majority of smartphone users globally [2]. This increase in smartphone usage led to the addiction behaviour to these devices, especially by 50 % of teens, as survey reports suggested [3].

Despite the structure and the design of smartphones that allow both hands, the use of single-handed is more preferred by young people [4]. The use of single-hand mainly relies on the thumb movement to reach for the keys for pressing, whereas, the rest of the hand is used for grasping [5]. It was reported that the average duration of smartphone usage among university students was > 3.5 hours/day, which was also accompanied by pain at the base of the thumb [6].

Complications and adverse effects of smartphones’ excessive usage may include dry eyes, computer vision problems, neck and shoulder problems, De Quervain’s tenosynovitis, and weakness of the thumb and wrist [7]. The thumb and wrist weakness is due to repetitive movement of flexion and extension over the wrist and fingers, which is increased with more duration spent over smartphones, eventually causing pain and fatigue [5, 8]. Further, this repetitive flexion and extension of the wrist are also known to be among the leading causes of carpal tunnel syndrome [9]. These complications would limit the hand’s functionality over time and may lead to psychological problems such as low quality of life [10].

The number of studies investigating the association between smartphone addiction/overuse and at least the hand-grip strength or pinch-grip strength is limited. A study compared hand-grip and pinch-grip strength between high-frequency smartphone users and low-frequency smartphone users among children [11]. The study reported that the higher frequency smartphone users had reduced hand and pinch-grip strength compared to the lower frequency smartphone users. Another study reported no difference in pinch-grip strength between high frequency and low-frequency smartphone users among young adults [12]. These two former studies utilized smartphone addiction scale- (short form) to group the participants based on the scale scoring. Smartphones have a weekly record of the duration of usage in units of time. To the best of knowledge, no study was found which investigated the association between smartphone usage (in units of time) and hand-grip, pinch-grip strength. Thus, the aim of the current study was to investigate the association between smartphone usage (in units of time) and hand-grip, pinch-grip strength among young people, and explore the factors associated with hand-grip, pinch-grip strength. It was hypothesized that both hand-grip and pinch-grip will be inversely associated with smartphone usage duration.

## Methods

### Participants

 One hundred participants from the Riyadh region, Saudi Arabia volunteered to participate in the study. Participants were approached in different and multiple locations for a single measurement. Inclusion criteria included using a smartphone to report the duration of weekly usage (using the iOS system), between 18 and 30 years old. Exclusion criteria included individuals with neuromuscular disorders, surgical history of median nerve release, tendon lesion of the thumb or hand, previous fracture of the hand or wrist, and any prior history of hand or wrist injury. According to the sample analysis performed using G*power software (version 3.1), a priori test for correlation showed that the minimum sample required to achieve the power of (1–β error probability) = 0.85 was 93 with the effect size (*d*) = 0.3.

### Procedure

**Fig. 1:**
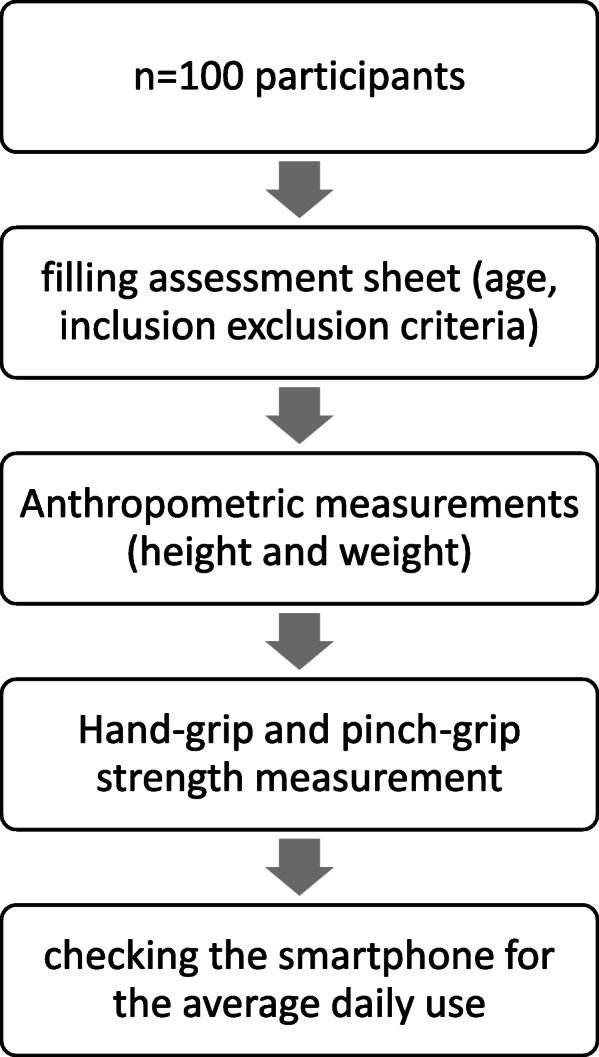
Flow diagram of the procedures of the study

Participants were invited verbally to volunteer to the study, or were approached on the spot (e.g. coffee shops, lounging areas) and were given information about the study. Upon acceptance to participate, informed consent was provided for signing, then the assessment sheet was filled by the investigator. Anthropometric measurement was obtained with minimal clothing. Height was measured using a portable Stadiometer to the nearest 0.5 cm (Holtain Ltd, Crosswell, UK). Weight was obtained via standing on a portable electronic zeroed scale (Wedderburn, Southampton, UK). Then, measurement of hand-grip and pinch-grip were performed, each for three times. Finally, the smartphone was checked for the weekly average screen duration (see Fig. 1). Participants were approached at the evening between the 5:00 pm and 8:00 pm. All testing was standardized to be carried out between these times of the day.

### Outcome variables

Hand-grip (hand-held) Jamar dynamometer (Sammons Preston Rolyan, Bolingbrook, IL, USA) was adjusted for the size of the hand of every participant via adjusting the handle of the grip to allow placement of the fingers at approximately 90° of flexion at the proximal and distal interphalangeal joints. Participants were requested to perform the test with their dominant hand. As recommended by the American Society of Hand Therapists [13], the hand-grip measurement was performed. Each participant was seated in an upright position, shoulder slightly abducted, elbow flexed at 90°, and wrist in a neutral position. Participants were instructed to squeeze the handle as much as possible three times, each time the squeeze lasted for 5 seconds and 30-seconds rest period was given between each time. Then, the average of the three trials in (kg) was recorded.

Pinch-grip dynamometer (Baseline, Fabrication Enterprises Inc., Irvington, NY, USA) position was similar to the position of hand-grip measurement. The same hand was used for doing the pinch-grip measurement. The gauge was placed between the tip of the thumb’s pad and the radial side of the middle phalanx of the index finger. Similarly, to the measurement of hand-grip, participants were instructed to pinch the gauge as much as possible three times, with 5-second duration for each, and 30-seconds rest period between each time. Again, the average of three trials in (kg) was recorded.

Smartphone usage duration was checked via asking the participant to check the screen time on the smartphone (which is an option available for all smartphones with the iOS system) for the average daily use in the past week. The average duration for smartphone screen time was calculated, and the average duration of smartphone daily usage for a week was reported in hours.

### Statistical analysis

Statistical analysis was performed using statistical package for social sciences (SPSS) (version 27, Chicago, IL, USA). Normality of the variables was tested using the Kolmogorov-Smirnov test. Normally distributed variables were presented as mean and standard deviation, and non-normally distributed variables were presented as median and interquartile range. Log transformation was performed on one single variable (Pinch-grip strength). To assess the relationship between smartphone usage and hand-grip pinch-grip strength (after log transformation), bivariate correlation using Pearson product-moment correlation analysis. Partial correlation correcting for weight and body mass index (BMI) as confounding variables was conducted to assess the same relationship between the study’s primary outcome variables. Two stepwise linear regressions were conducted to determine the factors associated with hand-grip and pinch-grip strength (correcting for age, weight, height, and BMI) where hand-grip and pinch-grip strength were the dependent variables and the smartphone usage duration was the independent variable. The level of significance was set at ≤ 0.05.

## Results

The demographic characteristics and the primary outcome variables of 100 participants are presented in Table 1. The average daily usage of smartphone among the participants was 7.8 ± 2.2.

**Table 1:** Characteristics and main outcome variables of *n* = 100 participants

Variable	value
Age (years)	21 (19–23)
Height (m)	1.67 ± 0.1
Weight (kg)	76.9 ± 16.7
BMI (kg/m^2^)	27.4 ± 4.8
*Outcome variables*	
Hand-grip strength (kg)	34 ± 7.8
Pinch-grip strength (kg)	6.7 (6–8.3)
Smartphone usage duration (h)	7.8 ± 2.2

Values are presented as means ± standard deviation, or median (25th to 75th percentile values) as appropriate. BMI, body mass index

### Correlation analysis

**Fig. 2:**
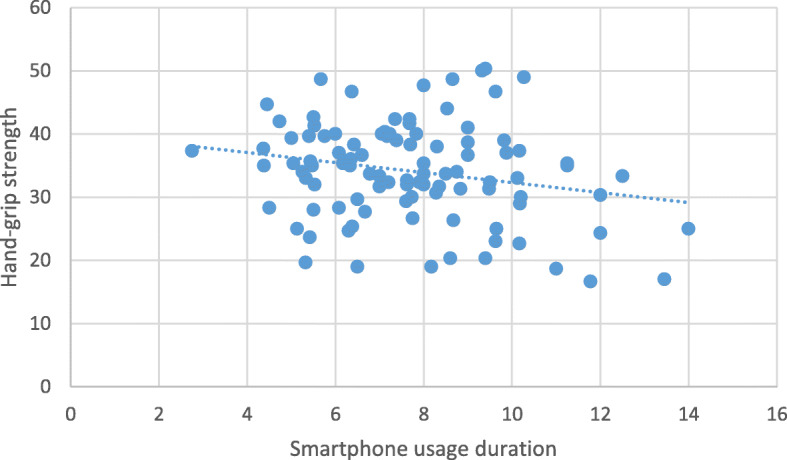
The relationship between smartphone usage duration and hand-grip strength

**Fig. 3:**
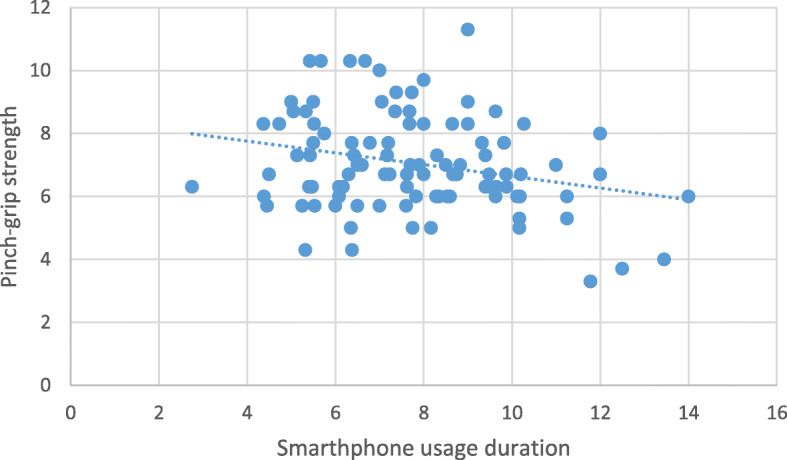
The relationship between smartphone usage duration and pinch-grip strength

Correlation analysis revealed a significant inverse relationship between smartphone usage duration and hand-grip strength and pinch-grip strength (Table 2). The more young people use their smartphones, the weaker their hand-grip (Fig. 2) and pinch-grip strength (Fig. 3). Further analysis revealed that there was a significant correlation between weight, and BMI with hand-grip strength (*r* = .22, *p* = .03; *r* = .27, *p* = .007 respectively). Thus, further correlation analysis was conducted with weight and BMI as a confounding variable. This analysis revealed that there was still a significant inverse relationship between smartphone usage duration and hand-grip strength (correcting for weight and BMI) (Table 2).

**Table 2:** Correlation analysis between smartphone usage duration and hand-grip, pinch-grip strength

Variable	Smartphone usage duration (hours)		Smartphone usage duration (hours) With weight and BMI as confounding variables	
	*r*	*p*	*r*	*p*
Hand-grip strength (kg)	-0.22	0.03	-0.22	0.03
Pinch-grip strength (kg)	-0.28	0.004	-0.27	0.008

### Linear regression

A stepwise linear regress was used to identify predictors and variables associated with the hand-grip and pinch-grip strength. A model which included age, height, weight, BMI as a confounding variable and the smartphone usage duration as an independent variable and hand-grip strength as a dependent variable was examined. Weight and height were excluded, and the remaining variables including age, BMI and smartphone usage duration explained 18.8 % of the variance in hand-grip strength (*F* (3, 96) = 7.4, *p* = .009, R^2^ = 0.188) (Table 3). For pinch-grip strength, the same model as in hand-grip strength was used. After analysis, weight, height, and BMI were excluded, and the remaining variables including age and smartphone usage duration explained 20.4 % of the variance in pinch-grip strength (*F* (2, 97) = 12.5, *p* < .001, R^2^ = 0.204) (Table 3). All the models were tested for collinearity, and no influence of collinearity in both models was found.

**Table 3:** Linear regression model for factors associated with hand-grip and pinch-grip strength

Variable	Hand-grip strength		Pinch-grip strength	
	β	t (*p)*	β	t (*p)*
Age	0.84	2.92 (0.004)	0.14	3.88 (< 0.001)
BMI	0.35	2.32 (0.02)		
Smartphone usage duration	-0.90	-2.70 (0.009)	-0.02	-3.67 (< 0.001)
R^2^ and *p* value of the model	R^2^	*p*	R^2^	*p*
	0.188	0.009	0.204	< 0.001

*BMI* body mass index

## Discussion

The current study explored the association between smartphone usage duration and hand-grip, pinch-grip strength among young people. There was a weak but significant inverse association between the variables. After using weight and BMI as a confounding factor, the inverse association remained significant. Additionally, linear regression revealed that variation in hand-grip and pinch-grip strength variation was explained by age and smartphone usage duration. The results may indicate that a longer duration of average daily smartphone usage was related and contributed to a weaker hand-grip and pinch-grip strength.

Many studies reported reduced hand function and multiple musculoskeletal problems when high-frequency smartphone users were compared against low-frequency smartphone users. A study compared median and ulnar nerve conduction velocity, forward head angle, neck pain, and hand-grip between two adolescents groups based on the duration of smartphone usage (group A which use smartphone < 4 hours/day vs. group B which use smartphone > 4 hours/day) [14]. The results showed that group B with a higher frequency of smartphone usage had weaker ulnar nerve conduction velocity, worse neck pain (on a visual analogue scale), and reduced forward head angle movement, whereas, no difference was found in hand-grip strength or median nerve conduction velocity. The grouping methodology in the former study was not reported, as it was unknown how the duration of smartphone usage was determined. Although group B had lower hand-grip strength than group A, the difference between the two groups was not significant [14]. Another study compared between two young adults groups over smartphones overuse (high, low) based on smartphone addiction scale with a non-smartphone users group as a control group, in which it was found that high-frequency smartphone users had enlarged median nerve, more pain at the thumb, decreased pinch strength and hand functions in comparison to low-frequency smartphone users [8]. Regarding the reduction of hand and pinch-strength and overall hand performance among children, similar results were reported among high-frequency smartphone users [11]. Majority of the former studies reported adverse effects of smartphone overuse over the hands, neck, median and ulnar nerve integrity. The question is whether these effects can be corrected or reduced with treatment or not.

Longitudinal studies investigating the effects of management to reduce the effect of smartphone overuse are limited. A study among 100 young adults investigated the effect of the exercise training program and postural correction on hand-grip strength, pinch-grip strength, upper extremity disability [15]. The study reported significant improvement in hand-grip strength, pinch-grip strength after a 12-week exercise program. The result of this study may indicate that increased smartphone usage is a factor that contributed to the weakness of the hand-grip and pinch-grip; hence, with training, these variables improved. Although the association between hand-grip strength and smartphone usage duration reported in the current study is weak, this may warrant further investigations in a larger sample with more control over other variables such as the level of physical activity and occupational factors.

The inverse association between smartphones usage duration and hand-grip pinch-grip strength can be explained due to the median nerve damage (enlargement), which was found to be associated with prolonged use of smartphones [8]. The median nerve controls the flexor-pronator muscles in the forearm and most of the musculature present in the radial portion of the hand which include the abduction of the thump, flexion of the hand and wrist, flexion of the digital phalanx of fingers [16]. The position of the hand and the thumb which is adapted while holding the smartphone for a prolonged duration may affect the median nerve [5]. Additionally, the repetitive flexion and extension of the wrist and excessive use of the thumb may also damage the median nerve [9]. Eventually, the damaged median nerve may lead to weakness of the muscles innervated by the median nerve, in which these muscles are the ones responsible for hand-grip and pinch-grip action.

It is worth noting that hand-grip strength measurement may induce muscular fatigue due to the repetition of multiple maximum efforts for a short period [17]. In this context, different types of fatigue can be mental or physical, including central or peripheral fatigue. The mental fatigue refers to the perception and cognition of fatigue which is related to the feeling of exhaustion after prolonged stress[18]. The central fatigue is described when there is a progressive reduction of motoneuronal output from the central nervous system to the muscles [19], whereas, peripheral fatigue is the result of changes in the neuromuscular junction that decreases the ability to generate the contractile force [18]. Hand-grip strength measurement could be influenced by the latter type. A study which investigated the effect of peripheral muscle fatigue during the testing of hand-grip strength showed a significant reduction throughout assessment which included three trials with 60-seconds interval [17]. In contrast, these findings were not confirmed in an earlier study with the same 60-seconds interval between the three trials [20]. In the current study, when the analysis was made between the three trials on hand-grip strength (data not reported), there was a significant difference between the mean of the three trials indicating a better result at the first trial. However, with regards to the pinch-grip strength, the same analysis was made which showed no difference in the mean between the three trials (data not reported). Therefore, the effect of peripheral fatigue on measurement of hand-grip and pinch-grip strength is still indecisive and further research is warranted in this area.

There are other factors that may affect the measurement of hand-grip and pinch-grip strength. For example, level of physical activity [21], participation in sports [22] and occupation [23] are common factors that can influence the hand muscles measurement. However, these factors’ influence has not been thoroughly examined especially the physical activity in young people. Majority of the previous studies did not find a significant impact of these factors over the hand muscles’ measurement. This is perhaps due to the lack of studies investigating the effect of exercise or physical activity on hand muscles [24]. Inclusion of these factors as variables in the current study with no doubt would give more details about the contributing factors to hand-grip and pinch-grip measurement in the present sample. This would be an aim for future investigations in the same area.

To the best of knowledge, this is the first study that utilized the technology provided by the smartphones (iOS system) which enable the users to monitor their average daily use of the smartphone in the form of weekly reports. This direct measurement of smartphone usage duration has been used before to examine the relationship between screen time over smartphone and sleep quality [25]. Although screen time can involve the duration of just merely looking at the screen, simple browsing over the phone will require the use of the thumb and the use of the palm to hold the smartphone (e.g. scrolling up and down over a page). Unlike filling a scale such as smartphone addiction scale, the current study method allows participants to self-monitor their daily average smartphones activity weekly without the need of filling scales. A study investigated the validity of self-reported measures about the time spent on smartphones compared to the time recorded on the log data from smartphones. Interestingly, it was found that people tend to overestimate their use of smartphones by 23 minutes more (on self-reports) than what is reported on their log data from smartphones [26]. The data obtained from smartphones has also been used to collect behavioural data in psychological sciences [27]. Thus, the use of data from smartphones is still emerging and may open additional ways to observe and detect abnormalities and pathologies.

Although the current study showed a weak relationship between smartphone usage duration and hand-grip, and pinch-grip strength, this study may add to the knowledge about the relationship between these variables. This relationship may open new ways to monitor the use of smartphones using objective approaches and further investigate other factors that may influence the strength of the hand’s muscles. There is a concern that young people these days tend to have a poor hand-strength as they are more accustomed to doing more texting and clicking than staying active and do some manual work [28]. Recommendations are suggesting larger studies to confirm relationships between smartphones overuse and muscles of the hand. The current study is shedding some light about this relationship which may help along with other future studies to establish educational strategies to overcome undetected adverse effects of prolonged use of smartphones, especially for the younger population. Further, the novelty in this study that using smartphone own monitoring system to detect the duration of the use of the phone may give a less time-consuming “more objective” option to use it instead of smartphone addiction scale.

In the current study, there was no control group to compare the participants’ results and investigate whether people with less time over their smartphones would have better hand-grip and pinch-grip strength. However, there are reference values for male and female regarding the hand-grip strength reported and consolidated in a descriptive meta-analytical study for different age groups [29]. The mean hand-grip reported in the current study was 34 (kg), and the age group of the participants was between 19 and 23 years old, in comparison to the references values for the same age group reported by Bohannon et al. [29], the participant in the current study had weaker hand-grip strength. However, there was no reference value found for pinch-grip strength. This may suggest that the participants in the current study indeed had a weak hand-grip strength.

There are some limitations to the study. Due to the cross-sectional design of the study, causality between variables should not be assumed. The study was gender-biased and conducted only on males due to cultural reasons. Therefore, the generalizability of the data is limited to males in the same age category. This further warrant research involving a female investigator and other age categories. Involvement of other daily tasks such as using keyboards on laptops may also influence hand-grip and pinch-grip strength. Thus, future research should also investigate other activities which involve the use of thumb and digitals of the hand during screening. Additionally, the screen use may include smartphone use for socializing, shopping, using maps while driving and observing favourite shows on the screen of the smartphone, which is included in the screen time. This may influence the representation of the time spent using the smartphone while using the hands and fingers. However, it is possible to think that socializing using smartphones is common in the age group representing the current study. Nevertheless, future studies need to report and separate between screen time and screen use. Furthermore, future studies should aim to address other confounding variables that may influence the hand-grip and pinch-grip measurement, including the level of physical activity, occupation or nature of the participants’ job.

In conclusion, the current study demonstrated an inverse association between prolonged use of smartphones with hand-grip and pinch-grip strength. The results indicated that more prolonged use of smartphones was weakly associated with weaker hand-grip and weaker pinch-grip. Future research should investigate the impact of prolonged use of smartphone on hand muscles in prospective longitudinal studies.

## Data Availability

The datasets used and/or analysed during the current study are available from the corresponding author on reasonable request.

## Abbreviations

SPSS: Statistical package for social sciences
BMI: Body mass index
